# Distal Radius Fractures: A Contemporary Review of Classification, Management, and the Evidence on Anatomy Versus Function

**DOI:** 10.7759/cureus.117977

**Published:** 2026-10-15

**Authors:** Jayanta K Laik, Ashutosh Mishra, Ritesh Kumar, Rajesh Thakur, Asmita D Laik, Mridul Ghosh

**Affiliations:** 1 Department of Joint Replacement and Orthopedics, Tata Main Hospital, Jamshedpur, IND; 2 Department of Orthopedics, Manipal Tata Medical College, Jamshedpur, IND; 3 Department of Joint Replacement and Orthopedics, Manipal Tata Medical College, Jamshedpur, IND; 4 Department of Biochemistry, Manipal Tata Medical College, Jamshedpur, IND

**Keywords:** distal radius fracture, fracture classification, kirschner wire fixation, orthopedic trauma, osteoporotic fracture, patient-reported outcomes, volar locking plate

## Abstract

Distal radius fractures (DRFs) are among the most common injuries in orthopedic practice and exhibit a bimodal age distribution, comprising high-energy injuries in younger patients and low-energy, osteoporosis-related fractures in older adults. Many heal well with closed reduction and cast immobilization, but a substantial proportion are unstable or displaced, prompting consideration of surgery. Over the past two decades, volar locking plate (VLP) fixation has become the dominant operative technique. However, randomized trials and recent meta-analyses consistently show that superior radiographic restoration does not reliably translate into superior patient-reported functional outcomes and that this discrepancy may become more pronounced with age. Many older adults with lower functional demands gain no clinically meaningful functional advantage from surgery, whereas younger, higher-demand patients and those with substantially displaced intra-articular fractures may benefit. Current guidelines therefore treat 65 years as a pragmatic threshold beyond which the functional benefits of surgery tend to diminish. This review summarizes the relevant anatomy, the evolution of classification systems, evaluation and imaging, comparative treatment evidence, management in older adults, complications, and rehabilitation.

## Introduction and background

Fractures of the distal radius are among the most frequently encountered injuries in orthopedic, hand, and emergency practice, and they impose a considerable burden through pain, temporary loss of function, and rehabilitation needs [1]. Their epidemiology is bimodal. A first peak occurs in children and younger adults, typically resulting from high-energy mechanisms such as falls from height, sports injuries, and road traffic trauma. A second and larger peak occurs in older adults, particularly postmenopausal women, in whom a low-energy fall onto an outstretched hand is sufficient to fracture osteoporotic bone [1,2]. A distal radius fracture (DRF) in an older patient therefore often serves as a sentinel event that should prompt assessment of bone health [1].

The clinical importance of these injuries lies not only in their frequency but also in the variability of their behavior. Some are stable and heal predictably with brief immobilization; others are unstable, intra-articular, or associated with neurovascular injury and carry a real risk of malunion, stiffness, and disability if inadequately managed [3]. Treatment has shifted markedly toward operative fixation, particularly volar locking plate (VLP) fixation, driven by the appeal of anatomical restoration and early mobilization [3]. Yet a recurring theme in the modern evidence is that the relationship between radiographic restoration and patient-perceived function is imperfect and that high-quality trials have tempered enthusiasm for routine surgery in lower-demand patients [3].

This review provides a practical, evidence-informed overview of DRFs for the practicing clinician. It covers functional anatomy and radiographic parameters, the evolution of classification, evaluation and imaging, the comparative evidence on nonoperative and operative treatment, management in older adults, complications, and rehabilitation.

## Review

Functional anatomy and radiographic parameters

The distal radius is the principal load-bearing articulation of the wrist. It transmits most of the axial load across the radiocarpal joint, with the remainder borne by the ulna through the triangular fibrocartilage complex (TFCC). Because restoration of normal architecture is the goal of reduction, three radiographic measurements are routinely assessed: radial inclination (normally 23.6°±2.5°), radial height (about 11.6±1.6 mm), and volar tilt (about 11.2°±4.6°) (Figure 1) [4]. Ulnar variance (normally 0.6±0.9 mm) is a further key parameter, since radial shortening that produces positive ulnar variance is associated with ulnar-sided wrist pain and impaired function [4].

**Figure 1:**
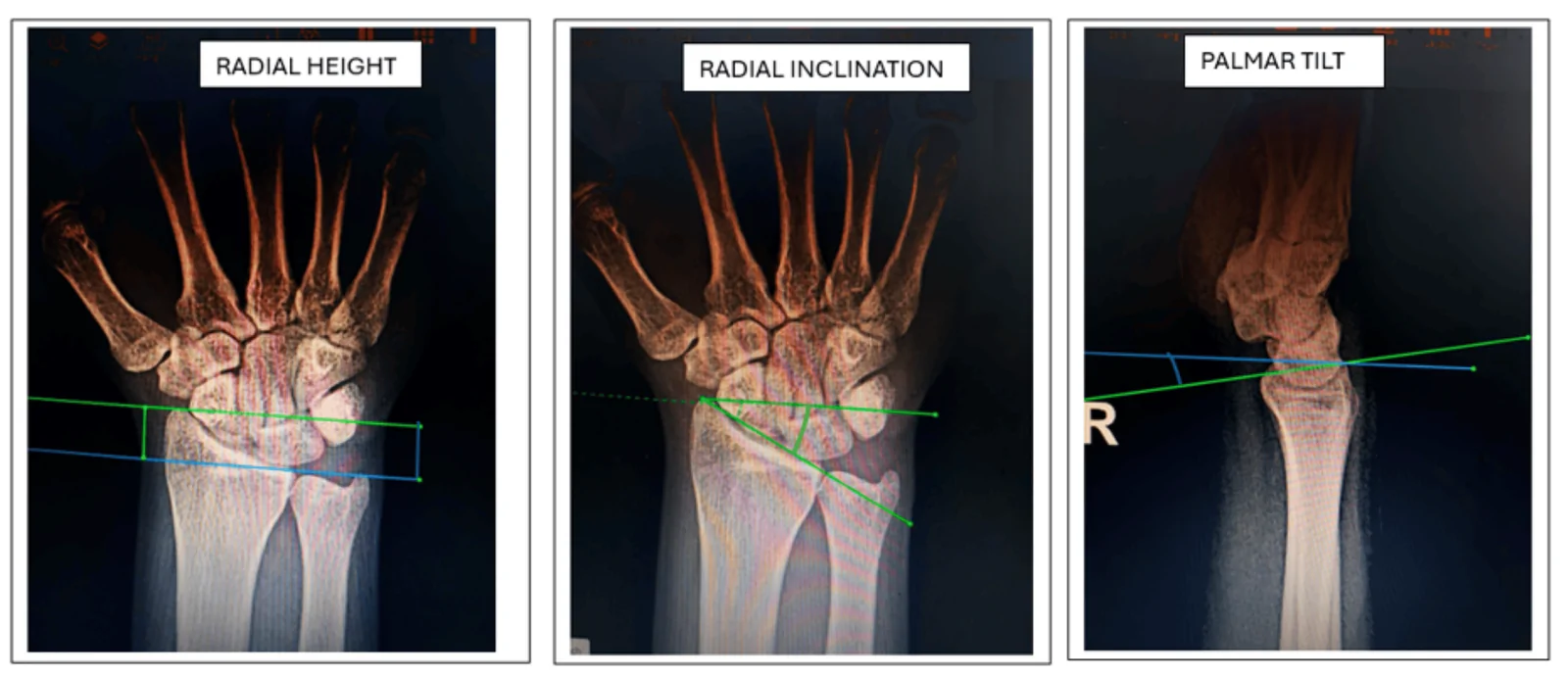
Standard radiographic parameters of the distal radius On the posteroanterior view, radial inclination (normally 23.6°±2.5°) is a measure of the radial slope, and radial height (approximately 11.6±1.6 mm) is the difference in axial length between the radial styloid tip and the ulnar corner of the distal radial articular surface. Ulnar variance denotes the relative length of the distal ulna with respect to the ulnar corner of the distal radial articular surface. On the lateral view, volar (palmar) tilt (approximately 11.2°±4.6°) is the angle formed between a line connecting the volar and dorsal rims and a line perpendicular to the longitudinal axis of the radial shaft. Restoration of these parameters is the goal of reduction. Attribution and Permission: Adapted with permission from Medoff [4]. © 2005 Elsevier Inc.

The distal radius may be conceptualized as three columns, including a radial column (radial styloid and scaphoid fossa), an intermediate column (lunate fossa and sigmoid notch), and an ulnar column (distal ulna and TFCC) [5]. This model aids fixation planning because independent fragments in different columns may each require support. The distal radioulnar joint (DRUJ), stabilized mainly by the TFCC and radioulnar ligaments, is integral to forearm rotation. Its integrity should be assessed deliberately in every DRF, both at presentation and again after fixation, because unrecognized or persistent DRUJ instability is one of the more common reasons for a poor functional result despite otherwise satisfactory bony healing [5].

Mechanism of injury and injury patterns

The dominant mechanism is a fall onto an outstretched, dorsiflexed hand. This generates a dorsally directed bending moment that typically produces dorsal displacement (the Colles pattern). Volar displacement (the Smith pattern) results from a fall onto a flexed wrist or a direct volar force, while volar or dorsal rim shear injuries (Barton fracture-dislocations) are intra-articular fracture-dislocations of the radiocarpal joint. The energy of injury shapes the fracture pattern. Low-energy falls in osteoporotic bone tend to produce metaphyseal comminution with relatively simple articular involvement, whereas high-energy trauma in younger patients may produce complex articular fragmentation, greater soft-tissue injury, and associated carpal or forearm injuries [1,3]. Recognizing the mechanism helps anticipate associated injuries, including scaphoid fracture, scapholunate ligament disruption, DRUJ injury, and acute median nerve compression.

Evolution of classification systems

Classification of DRFs has evolved over more than two centuries, and each system reflected and advanced the prevailing goals of management. Early eponymous descriptions provided a shared vocabulary for the direction of displacement and guided closed reduction. The Frykman classification then drew attention to intra-articular and DRUJ involvement; the Melone classification highlighted the four-part intra-articular “medial complex” of the lunate fossa and the need for articular reconstruction; the Fernandez classification linked injury mechanism to a treatment strategy; and the Arbeitsgemeinschaft für Osteosynthesefragen/Orthopedic Trauma Association (AO/OTA) system introduced a standardized alphanumeric grading of severity that underpins research, audit, and implant planning (Figure 2) [6-9]. This progression is summarized in Table 1.

**Figure 2:**
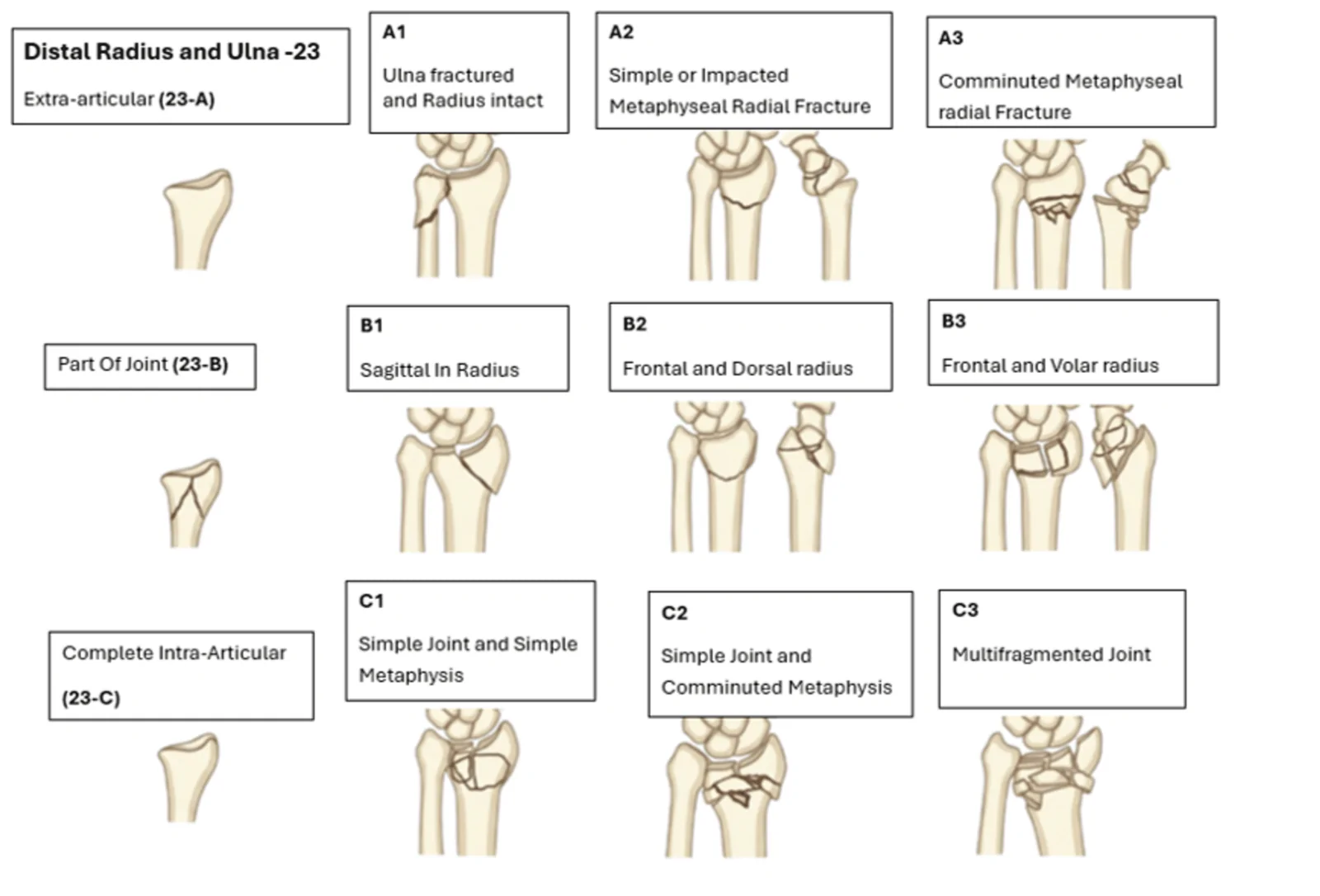
Schematic of the AO/OTA classification of DRFs: type A, extra-articular; type B, partial articular (part of the articular surface remains in continuity with the shaft); and type C, complete articular (the articular surface is disrupted and separated from the shaft), with increasing complexity from type A to type C DRF: distal radius fracture; AO: Arbeitsgemeinschaft für Osteosynthesefragen; OTA: Orthopedic Trauma Association Attribution and Permission: Adapted with permission from Müller et al. [10]. © 1990 Springer-Verlag.

**Table 1:** Evolution of the principal DRF classification systems and their contribution to management DRUJ: distal radioulnar joint; DRF: distal radius fracture

Classification (era introduced)	Basis	Principal contribution to management
Eponymous (Colles, Smith, Barton; 19th century)	Direction and pattern of displacement	Provided a common language; guided closed reduction and splinting
Frykman (1967)	Radiocarpal and/or radioulnar articular involvement plus ulnar styloid fracture [6]	Drew attention to articular and DRUJ involvement; early prognostic intent
Melone (1984)	Four-fragment model of the intra-articular surface (medial complex) [7]	Emphasized the lunate fossa die-punch fragment and articular reconstruction
Fernandez (1993)	Injury mechanism: bending, shear, compression, avulsion, or combined mechanisms [8]	Linked mechanism to a surgically oriented treatment strategy
AO/OTA (1990s; compendium updated 2018)	Alphanumeric severity: extra-articular (A), partial articular (B), complete articular (C) [9]	Standardized severity grading for research, audit, and implant selection

Each system improved how surgeons describe fractures, communicate about them, and plan reconstruction, and collectively, these systems contributed to the increasingly operative management of DRFs in recent decades. Two caveats temper this progress. First, most systems show only moderate interobserver and intraobserver reliability, and none reliably predicts outcomes in isolation [3,9]. Second, this taxonomic and radiographic sophistication does not appear to have translated proportionately into better patient-reported function, an observation examined in the sections that follow.

Clinical evaluation and imaging

Assessment begins with the history, including the mechanism and energy of injury, hand dominance, occupation and functional demands, comorbidities, and bone-health risk factors. Examination documents deformity, swelling, skin integrity, and the neurovascular status of the hand. Acute median nerve dysfunction is the most common neurological complication and should be actively sought, since progressive or severe symptoms may mandate urgent reduction or carpal tunnel release [3]. The DRUJ, elbow, and scaphoid should also be examined. Posteroanterior and lateral radiographs are the mainstay of imaging and allow measurement of radial inclination, radial height, volar tilt, ulnar variance, and articular step-off or gap [4]. Computed tomography is indicated for intra-articular fractures when the degree of articular displacement or fragment configuration is not adequately defined on plain films [3]. Three-dimensional CT reconstruction is increasingly used for complex articular fractures because it improves characterization of fragment configuration and can influence both fracture classification and the operative plan [11]. Magnetic resonance imaging is not routinely performed but may be used selectively for suspected ligamentous or TFCC injury.

Stability and acceptable reduction

Distinguishing stable from unstable fractures guides management, since stability determines whether an acceptable position is likely to be maintained without surgery. Predictors of instability include the initial degree of dorsal angulation and displacement, dorsal metaphyseal comminution, intra-articular involvement, radial shortening, an associated ulnar fracture, and advanced age with poor bone quality [12,13]. The criteria proposed by Lafontaine and colleagues in 1989 remain historically important and are frequently cited as an early framework for identifying potentially unstable fractures [12]. The importance of articular reduction derives largely from the work of Knirk and Jupiter, who linked residual intra-articular incongruity with later post-traumatic arthritis in young adults [14]. This principle retains validity in young, high-demand patients, but its modern application is more nuanced. Later studies have questioned the strength of this association: long-term follow-up shows that radiographic post-traumatic arthritis after intra-articular fracture is common yet frequently asymptomatic, correlating only weakly with pain and function [15]. Commonly cited thresholds for acceptable alignment, recognized as guidelines rather than absolute cutoffs, include dorsal tilt within about 10° of neutral, radial shortening under about 2 mm-3 mm, restoration of radial inclination toward the normal anatomical range of approximately 21°-25°, and articular step-off or gap under 2 mm in higher-demand patients [3,4]. Tolerance for residual deformity is generally greater in low-demand older patients, in whom malunion is often well tolerated [3].

Non-operative management

For stable fractures and for displaced fractures that can be adequately reduced and are expected to remain stable, closed reduction and immobilization remain the standard [3,16]. Reduction is performed under adequate analgesia using traction and manipulation to reverse the deforming force, followed by a well-molded cast or splint; a below-elbow cast suffices for most fractures. Serial radiographic follow-up during the first few weeks is important to detect secondary displacement, which may prompt reconsideration of surgery [3,16]. A Cochrane review of conservative interventions found limited high-quality evidence favoring any single method of reduction, cast type, or position, indicating that meticulous technique and vigilant follow-up matter more than the specific regimen [16].

Operative management

Indications and Techniques

Surgery is considered for fractures that are irreducible, unstable after reduction, significantly displaced and intra-articular, or associated with injuries requiring operative attention, as well as for higher-demand patients in whom anatomical restoration is prioritized [3]. Percutaneous Kirschner-wire (K-wire) fixation is a minimally invasive, low-cost technique suited to extra-articular and simple fracture patterns with adequate bone quality, often supplemented by a cast; its limitations include less rigid fixation and pin-site complications. External fixation, which relies on ligamentotaxis, retains value in open or highly comminuted fractures and as a temporizing measure, although its use has declined; a Cochrane review found no clear superiority over conservative treatment across unselected fractures [17]. VLP fixation has been the dominant technique since the early 2000s, providing stable subchondral support through a volar approach and allowing early mobilization across a broad range of fracture patterns [3]. Dorsally displaced fractures with dorsal comminution or discrete dorsal fragments may be treated with low-profile dorsal or fragment-specific implants. Although contemporary designs have reduced the historically high rates of extensor tendon complications, implant removal remains more frequent following dorsal plating than following volar plating, reflecting the continued potential for hardware-related extensor tendon irritation and symptoms [3,5]. Fragment-specific fixation, which stabilizes individual columns and fragments with dedicated low-profile implants, is best reserved for complex fracture patterns that a single volar plate cannot capture. A randomized trial found that, compared with VLPs, fragment-specific fixation achieved equivalent patient-reported outcomes but a substantially higher complication rate, so its role is targeted rather than routine [18]. Wrist arthroscopy can assist in the reduction of displaced articular fragments and the assessment of soft-tissue injury; guideline evidence supports its selective use rather than routine application [3].

Comparative Evidence on Fixation Technique

High-quality randomized evidence has repeatedly failed to show clear superiority of any single technique in the medium term. The UK Distal Radius Acute Fracture Fixation Trial (DRAFFT) randomized adults with dorsally displaced fractures to K-wire or VLP fixation and found no clinically relevant difference in the Patient-Rated Wrist Evaluation (PRWE) scores at three, six, or twelve months, with K-wire fixation being less costly [19]. The DRAFFT2 trial compared K-wire fixation with molded cast immobilization and found no meaningful difference in wrist function at twelve months, although about 13% of patients treated with a cast later required surgery because of loss of reduction [20]. A recent systematic review and meta-analysis concluded that surgery is associated with statistically better PRWE and Disabilities of the Arm, Shoulder and Hand (DASH) scores in adults overall, although whether these differences are clinically meaningful remains debatable [21]. These findings support the guideline position that, beyond about three months, no consistent difference in clinical or radiographic outcomes is attributable to fixation technique and that the choice should be guided by fracture pattern, patient factors, resources, and surgeon expertise rather than by the presumption that more complex fixation yields better results [3,19].

Anatomical Restoration and Its Limits

A consistent signal across this literature is that improvements in classification and radiographic restoration have not been matched by proportionate gains in patient-reported function. Table 2 summarizes pooled functional data by age group from a recent meta-analysis [21].

**Table 2:** Pooled functional and clinical outcomes of operative versus nonoperative management, as reported in the systematic review and meta-analysis by Bell et al. For the DASH and PRWE, lower scores denote better function, so a negative MD favours surgery; a complication OR below 1.0 favours surgery but is non-significant here. The MCID is approximately 10 points for the DASH and about 11 points for the PRWE [22]. DASH: Disabilities of the Arm, Shoulder and Hand; PRWE: Patient-Rated Wrist Evaluation; MCID: minimal clinically important difference; MD: mean difference; OR: odds ratio Data adapted with permission from Bell et al. [21].© 2025 The British Editorial Society of Bone & Joint Surgery.

Functional/clinical outcome	Cohort/subgroup	Follow-up	Pooled effect estimate (95% CI)	Effect magnitude vs. MCID threshold	Clinical significance
Upper limb function (DASH)	Adult cohort (≥18 yrs)	3 months	MD -10.58 (-13.15 to -8.01)	Approaches DASH MCID (~10.8)	Early statistical advantage favoring surgical fixation
Upper limb function (DASH)	Adult cohort (≥18 yrs)	12 months	MD -4.17 (-6.05 to -2.30)	Below MCID threshold	Statistically significant; not clinically meaningful long-term
Wrist-specific function (PRWE)	Elderly subgroup (≥65 yrs)	3 months	MD -8.53 (-18.13 to 1.07); p=0.08	Below PRWE MCID (11.5)	No statistically significant difference
Wrist-specific function (PRWE)	Elderly subgroup (≥65 yrs)	12 months	MD -2.13 (-4.60 to 0.33); p=0.09	Below PRWE MCID (11.5)	Equivalent functional outcomes between arms
Adverse events	Elderly subgroup (≥65 yrs)	12 months	OR 0.73 (0.21 to 2.49); p=0.62	No difference	Comparable overall complication profile

The pattern is age-dependent. In adults overall, surgery confers an early functional advantage that is statistically robust but, by 12 months, falls below the threshold generally considered clinically meaningful. In patients aged 65 years and older, no significant or clinically meaningful functional advantage remains at any time point, and complication rates do not differ [21]. This disconnect between anatomy and function, which some authors have described as a “radiographic trap”, should be interpreted cautiously, given the heterogeneity of the pooled data. However, it suggests that anatomical success predicts patient-reported outcomes less reliably than has traditionally been assumed, particularly in older patients [21,23]. The practical implication is not that anatomical reduction is unimportant, but that its pursuit is best calibrated to the individual: rigorous where meaningful function depends on it, and more permissive where malunion is well tolerated, and the risks and costs of surgery may outweigh its marginal benefit [14,24]. These considerations are summarized in Table 3.

**Table 3:** Factors shifting the balance toward versus away from operative management. These are considerations to be weighed together; no single factor is decisive DRUJ: distal radioulnar joint; AO: Arbeitsgemeinschaft für Osteosynthesefragen Data compiled from references [14,24].

Domain	Shifts toward surgery	Shifts toward non-operative care
Patient demand and physiology	Younger, high-demand, dominant-hand injury; fit for surgery and rehabilitation	Older (≥65 years), lower demand; frailty or high anesthetic risk
Fracture pattern	Substantially displaced intra-articular fracture (AO type C); articular step or gap ≥2 mm	Extra-articular or minimally displaced; acceptable articular congruity
Stability and reducibility	Unstable (dorsal comminution, radial shortening) or irreducible; alignment lost on serial radiographs	Stable, or adequately reduced and holding within acceptable parameters
Associated injuries	Open fracture; acute or progressive median nerve compromise; DRUJ instability requiring fixation	No associated injury requiring operative treatment
Bone quality	Adequate for durable fixation purchase	Very poor bone stock (relative), where malunion is likely to be well tolerated
Context and patient values	Prioritizes early recovery; reliable access to surgery and therapy	Prefers to avoid surgery; limited access to follow-up or rehabilitation

Management in older adults

Management in older adults is among the most debated questions in the field because this group sustains most DRFs and differs in functional demands, bone quality, and comorbidities. Physiological age, pre-injury functional status, activity level, independence, cognitive status, comorbidity burden, and patient preferences should also be considered. A landmark randomized trial by Arora and colleagues in patients aged 65 years and older found that VLP fixation produced superior radiographic parameters and modestly better early grip strength, but differences in pain and patient-reported function had largely disappeared by 12 months, while the operative group had more complications [24]. A secondary analysis of the Wrist and Radius Injury Surgical Trial (WRIST) reinforced this observation, showing that the degree of anatomical restoration did not reliably predict patient-reported outcomes in older adults [23,25]. Two further trials support a similar conclusion. The volar plating versus casting of unstable distal radius fractures in patients older than 65 years (VOLCON) trial found comparable functional outcomes with VLP fixation and casting for unstable fractures in patients older than 65 years, despite better radiographic restoration with surgery [26]. The Distal Radius Fracture Trial (DRIFT), which addressed fractures that lose alignment during nonoperative treatment, found a statistically significant advantage for fixation on the PRWE, but the magnitude of this advantage fell below the prespecified threshold for clinical importance [22].

The evidence is not uniformly against surgery, and a balanced interpretation is essential. The Displaced Intra-Articular Type C Distal Radius Fractures Trial (DART) tested whether casting was noninferior to surgery in patients aged 65 years and older with substantially displaced intra-articular (AO type C) fractures. In the primary intention-to-treat analysis, noninferiority of casting was not demonstrated, with a one-year PRWE score of 20.4 in the casting group versus 14.5 in the surgical group. The absolute difference of about six points is modest and arguably at or below the threshold for clinical importance. As-treated analyses were more favorable to casting; therefore, the trial is best interpreted as indicating that a subgroup of patients with substantially displaced intra-articular fractures may benefit from surgery rather than as providing a general endorsement of fixation [27]. A three-year follow-up of a randomized trial in patients older than 70 years similarly reported a modest but persistent functional advantage for VLP fixation in selected patients [28]. A meta-analysis focusing on older adults found that, whereas surgery improved functional scores across adults overall, there was no significant or clinically meaningful benefit in patients aged 65 years and older [21].

Reflecting this evidence, the 2020 American Academy of Orthopedic Surgeons/American Society for Surgery of the Hand (AAOS/ASSH) clinical practice guideline uses 65 years as a pragmatic proxy for functional activity, below which patients are more likely to benefit from surgery and above which the benefits of surgery relative to nonoperative management tend to diminish [3]. The guideline favors an individualized decision incorporating functional demands, expectations, comorbidities, and patient values; recommends selective use of supervised therapy, arthroscopic assistance, and routine repeat radiographs; and endorses multimodal, opioid-sparing analgesia [3].

Complications

Complications span neurological, tendinous, articular, and functional domains. Median nerve dysfunction, ranging from transient neurapraxia to acute carpal tunnel syndrome, is the most frequent neurological complication and may require urgent decompression when progressive or severe [3]. Tendon complications include rupture of the extensor pollicis longus, which can occur even after nondisplaced fractures treated nonoperatively, and flexor or extensor tendon attrition related to implant prominence after plating [3]. Malunion, most often involving dorsal angulation with radial shortening, can cause pain, weakness, reduced motion, and DRUJ dysfunction and may require corrective osteotomy in symptomatic patients [3]. Complex regional pain syndrome, persistent stiffness, and DRUJ instability are further recognized complications. A systematic review of unstable fractures in older adults confirms that both operative and nonoperative pathways carry distinct complication profiles, reinforcing that the balance of harms as well as benefits should inform treatment [29]. Pin-site problems with wires and hardware-related soft-tissue irritation with plates are reduced by careful technique, appropriate implant selection, and accurate placement [3].

Rehabilitation and functional recovery

Rehabilitation aims to restore range of motion, grip strength, and functional hand use while protecting healing. From the outset, early active finger and thumb motion, limb elevation, and edema control are emphasized to prevent stiffness and swelling, while tendon-gliding exercises help reduce adhesions. Wrist mobilization is then progressed according to fracture stability and fixation method, earlier after rigid internal fixation and later after cast immobilization. Progressive grip and resistance strengthening are generally deferred until early bony union, at around six weeks, and advanced gradually thereafter as healing and symptoms permit. Evidence does not strongly support routine formal supervised therapy for all patients; current guidance favors a home exercise program for many, with supervised hand therapy reserved for patients with specific deficits or greater complexity [3]. Realistic counseling about the recovery timeline, recognizing that pain and function may recover at different rates and that maximal improvement can take several months, supports patient satisfaction and engagement.

Bone health and secondary prevention

In older adults, a low-energy DRF is an important indicator of osteoporosis and an elevated risk of future fractures, including hip fractures. The acute injury therefore provides an opportunity for secondary prevention through bone mineral density assessment, falls risk evaluation, and initiation of bone-protective therapy when indicated [1]. Integrating fracture care with bone health pathways, such as fracture liaison services, is increasingly emphasized.

## Conclusions

Distal radius fractures are common, heterogeneous injuries whose management depends on the interaction between fracture pattern and patient factors rather than on any single technique. Restoration of key radiographic parameters remains the goal of reduction, but randomized evidence indicates that radiographic superiority does not consistently translate into better patient-reported function, particularly in older, lower-demand patients. Nonoperative management with careful reduction and vigilant follow-up remains appropriate for stable and adequately reducible fractures, while operative fixation, most commonly VLP fixation, with K-wire and other techniques retaining defined roles, is reserved for unstable, irreducible, or significantly displaced intra-articular fractures and for higher-demand patients. In older adults, current evidence supports individualized decision-making anchored on functional demands rather than a rigid age threshold, while recognizing that some subgroups, such as those with substantially displaced intra-articular fractures, may still benefit from surgery. Vigilance for complications, attention to bone health, appropriately tailored rehabilitation, and realistic counseling together underpin optimal outcomes.
